# Identification of Candidate Genes for Clubroot-Resistance in *Brassica oleracea* Using Quantitative Trait Loci-Sequencing

**DOI:** 10.3389/fpls.2021.703520

**Published:** 2021-11-16

**Authors:** Fuquan Ce, Jiaqin Mei, Haiyan He, Yu Zhao, Wenhui Hu, Fengqun Yu, Qinfei Li, Xuesong Ren, Jun Si, Hongyuan Song, Wei Qian

**Affiliations:** ^1^College of Horticulture and Landscape Architecture, Southwest University, Chongqing, China; ^2^College of Agronomy and Biotechnology, Southwest University, Chongqing, China; ^3^Key Laboratory of Horticulture Science for Southern Mountains Regions, Ministry of Education, Chongqing, China; ^4^Academy of Agricultural Sciences, Southwest University, Chongqing, China; ^5^State Cultivation Base of Crop Stress Biology for Southern Mountainous Land of Southwest University, Chongqing, China; ^6^Chongqing Key Laboratory of Olericulture, Chongqing, China; ^7^Agriculture and Agri-Food Canada, Saskatoon Research and Development Center, Saskatoon, SK, Canada

**Keywords:** *Brassica oleracea*, *Plasmodiophora brassicae*, clubroot, genomic resequencing, candidate gene, functional marker

## Abstract

Clubroot caused by *Plasmodiophora brassicae* is a devastating disease of cabbage (*Brassica oleracea*). To identify quantitative trait loci (QTLs) for clubroot resistance (CR) in *B. oleracea*, genomic resequencing was carried out in two sets of extreme pools, group I and group II, which were constructed separately from 110 and 74 F2 cloned lines derived from the cross between clubroot-resistant (R) cabbage “GZ87” (against race 4) and susceptible (S) cabbage “263.” Based on the QTL-*sequencing* (QTL-Seq) analysis of group I and group II, three QTLs (i.e., *qCRc7-2, qCRc7-3*, and *qCRc7-4*) were determined on the C07 chromosome. RNA-Seq and qRT-PCR were conducted in the extreme pools of group II before and after inoculation, and two potential candidate genes (i.e., *Bol037115* and *Bol042270*), which exhibiting upregulation after inoculation in the R pool but downregulation in the S pool, were identified from the three QTLs on C07. A functional marker “SWU-OA” was developed from *qCRc7-4* on C07, exhibiting ∼95% accuracy in identifying CR in 56 F2 lines. Our study will provide valuable information on resistance genes against *P. brassicae* and may accelerate the breeding process of *B. oleracea* with CR.

## HIGHLIGHTS

- QTLs and potential candidate genes for clubroot resistance were identified in *Brassica oleracea*.

## Introduction

Clubroot disease caused by the obligate parasite *Plasmodiophora brassicae* is a devastating disease that affects *brassica* species worldwide including important crops such as *Brassica oleracea*, *Brassica rapa*, and *Brassica napus* (Dixon, 2009; Chu et al., 2014). The pathogen usually causes gall formation on plant roots, which may subsequently hamper the uptake of sufficient nutrients and water, leading to abnormal growth of plants, and finally resulting in severe yield losses and economic damage to the crop (Devos et al., 2005). It is hard to control this disease once a field is contaminated with the pathogen since the resting spores of the soil-borne pathogen can survive in the soil for as long as 20 years (Chu et al., 2014). Cultural management and chemical fungicides are currently the common approaches to control clubroot (Friberg et al., 2005; Ludwig-Müller et al., 2009; Peng et al., 2015), but the efficiency is unstable and the environmental damage from fungicides cannot be ignored. Therefore, developing resistant (R) cultivars is the most effective, economical, and environment-friendly way to control clubroot.

Identification of clubroot resistance (CR) loci is the base of resistance breeding of *Brassica* crops. In *B. rapa*, the CR traits are reported to be controlled in a qualitative (Kuginuki et al., 1997) and quantitative manner (Suwabe et al., 2006) depending on genotypes studied, and at least 14 pathotype-specific loci have been identified from various European fodder turnips, such as *CRa, CRb, CRb^*Kato*^*, *CRc, CRd, CRk, Crr1a, Crr1b, Crr2, Crr3, Crr4, CrrA5, CRs, PbBa1.1, PbBa3.1, PbBa3.3, PbBa8.1, PbBrA08^*Banglin*^, qBrCR38-1, qBrCR38-2, Rcr1, Rcr2, Rcr3, Rcr4, Rcr5, Rcr8*, and *Rcr9* (Chen J. et al., 2013; Yu et al., 2016, 2017; Huang et al., 2017, 2019; Chang et al., 2019; Zhu et al., 2019; Karim et al., 2020; Mehraj et al., 2020). *Brassica* species containing the B-genome are a useful source of genes when breeding for disease resistance (Navabi et al., 2013). A race-specific CR gene (*Rcr6*) was identified in *Brassica nigra* (Chang et al., 2019). In *B. napus*, more than 30 CR loci and some dominant CR genes have been proposed in the A and C genome, such as *Pb-Bn1, BnA04, BnC05, PbBn-a-1, PbBn-Korp-1, SCR-C4a, SCR-C4b, BnC02_0414, BnC07_0238*, etc., (Manzanares-Dauleux et al., 2000; Werner et al., 2007; Li et al., 2016; Hejna et al., 2019). In *B. oleracea*, a few quantitative trait loci (QTLs) were identified, including *Pb3*, *Pb4*, *PbBo1*, *Pb-Bo (Anju) 1*, *Pb-Bo (Anju) 2*, *Pb-Bo (Anju) 3*, *Pb-Bo (Anju) 4*, *CRQTL-YC*, *CRQTL-GN_1*, and *CRQTL-GN_2* (Voorrips et al., 1997; Moriguchi et al., 1999; Rocherieux et al., 2004; Nagaoka et al., 2010; Lee et al., 2015; Dakouri et al., 2018), possibly due to the lack of R resource and the quantitative trait nature of CR in *B. oleracea* (Nagaoka et al., 2010). Interestingly, a CR gene (*Rcr7*) conferring strong resistance to clubroot was identified in *B. oleracea* (Dakouri et al., 2018).

In a previous study (Peng et al., 2018), several QTLs for traits, such as root length and *P. brassicae* content in roots, were identified from a hybrid cabbage (*B. oleracea* L. var. *capitata*) variety “GZ87,” which was R to *P. brassicae* race 4. However, QTLs for CR in “GZ87” have not been identified yet. In this study, QTL-sequencing (QTL-Seq) strategy (Takagi et al., 2013) was used to identify QTLs using genome resequencing in two sets of extreme pools. Potential candidate genes were identified from important QTL regions, and a functional marker was further developed for molecular-assisted selection. Our study will provide important information for the breeding of clubroot-R cabbage.

## Materials and Methods

### Pathogen Isolates

Inoculum (resting spores) were obtained from clubbed roots of infected plants collected from Fuling, Chongqing, China (E107.6459, N29.5709), where *P. brassicae* race 4 is the dominant pathogen causing clubroot. The clubbed roots were washed and stored at −20°C. Before inoculation, resting spores were extracted from clubbed roots by homogenizing the clubroot galls. The homogenate was filtered through a double gauze and then adjusted to a concentration of 4 × 10^7^ spores/ml for inoculation.

### Plant Materials and Resistance Test

**FIGURE 1:**
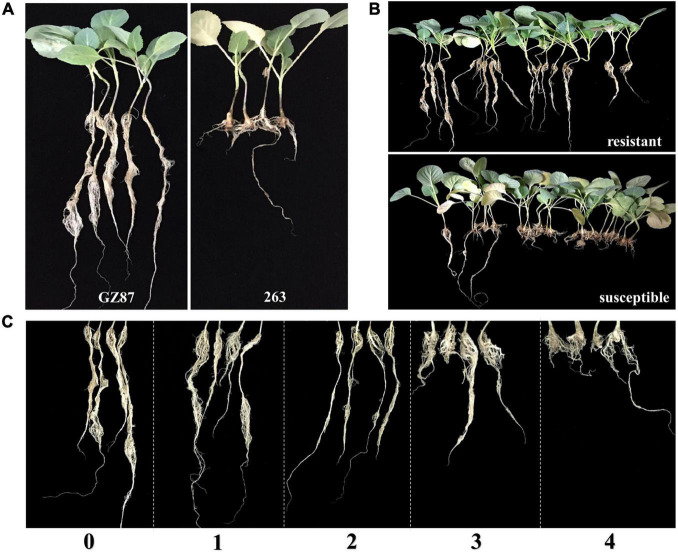
Phenotypes of the *Brassica oleracea* parents and F2 population. **(A)** Two cabbage lines, “GZ87” and “263”. **(B)** The phenotypes of resistant (R) and susceptible (S) plants in the F2 population. **(C)** The clubroot degree of the F2 population was counted with the scale criterion from 0 to 4. Plants were inoculated with race 4 of *Plasmodiophora brassicae*.

Two cabbage lines, “GZ87” (with CR to *P. brassicae* race 4) and “263” [susceptible (S) to *P. brassicae* race 4], were selected as parents to develop an F2 segregating population comprising 184 cloned lines through tissue culture following the asexual reproduction technology (Luo et al., 2000). Vegetative plants were transplanted into 72-well plug trays after rooting and kept in a phytotron (16/8 h light/dark cycle under 25 ± 2°C). The inoculation of *P. brassicae* was conducted 1 week after transplanting, in which each plant was inoculated with 5 ml *P. brassicae* resting spores suspension (4 × 10^7^ spores/ml) at the stem base using an irrigation method (Nagaoka et al., 2010; Peng et al., 2018). Notably, 15 plants of each line with three replications were inoculated. Plants were rated at 6 weeks after inoculation according to Chen J. et al. (2013) on a 0–4 scale (Figure 1C), where 0 = no clubs, 1 = less than three small clubs on the lateral roots, 2 = less than three large clubs on the lateral roots, or more small clubs, or small clubs on the taproot, 3 = large clubs on the taproot and lateral roots or many large clubs on the lateral roots, 4 = taproot rots, no lateral roots, or less. Disease index (DI) was calculated according to the following formula (Strelkov et al., 2006): DI = [(0n0 + 1n1 + … + 4n4) × 100]/(4 × NT), where n0–n4 are the numbers of different plants in different disease grades, and NT is the total number of plants tested. The group resistance grading standard described in the study by Liu et al. (2018) was followed: DI = 0, highly R; DI < 10, R; 10 ≤ DI < 20, moderately S; 20 ≤ DI ≤ 50, S; and DI > 50, highly S. Disease incidence (DIC) was recorded as the percentage of diseased plants in the total number of inoculated plants.

### Whole-Genome Resequencing and Quantitative Trait Loci-Seq Analysis

To improve the reliability of QTL mapping, the F2 segregating population was randomly divided into two small groups, in which group I and group II contained 110 and 74 F2 lines, respectively. Each of 17/20 extreme R and S lines was selected from group I/II. Genomic DNA was extracted from young leaves of two parents and the extreme lines following the cetyltrimethylammonium bromide (CTAB) method (Doyle, 1991). An R pool and an S pool were constructed within each group by mixing an equal volume of DNA from the extreme R and S lines. The two pools in group I and two parents were subjected to whole-genome resequencing on an Illumina HiSeq X Ten platform by using a biomarker (Beijing, China), while the two pools in group II and parents were sequenced on a NovaSeq 6000 platform by using the same service provider. Reads with low-quality bases (*Q* ≤ 20) or with an N ratio over 10% in the pooled samples and over 1% in parental lines were removed. Then, the high-quality reads were mapped to the reference genome of *B. oleracea*^1^ using BWA software (Li and Durbin, 2009). The reads filtering and single nucleotide polymorphisms/insertions or deletions (SNPs/indels) calling were conducted according to Zhou et al. (2015).

The QTL-Seq analysis was performed in accordance with the method of Takagi et al. (2013). The SNP-index is calculated for each SNP and relating SNP-index and chromosome positions are obtained for both the R and S pools separately. The two SNP-index are compared to identify the region with Δ(SNP-index)? value that is specific to the disease resistance. In brief, the Δ(SNP-index) was calculated for each locus (SNP or indel), and a sliding window analysis was applied to generate the Δ(SNP-index) curve with a window size of 100 Kb and increment of 10 Kb (Fekih et al., 2013). Significant SNP index values (the 1% right tail) were identified as the empirical thresholds, where the threshold value was 0.34 for samples in group I and 0.35 for samples in group II.

### Transcriptome Sequencing

The whole roots were collected from extreme lines in group II at 0, 4, 7, and 14 days after inoculation (DAI), with three biological replications. Roots from R and S lines were mixed at each time point, respectively, resulting in 24 samples (R0, R4, R7, R14, S0, S4, S7, and S14, three biological replications for each) for RNA extraction and transcriptome sequencing (RNA-seq). Total RNA was extracted from these pooled root samples using TRNzol-A + Reagent (TianGen, Beijing, China), and 24 library preparations were generated and sequenced on an Illumina Hiseq 2000^TM^ platform using a biomarker. Clean reads were aligned to the *B. oleracea* reference genome. Fragments per kilobase of transcript per million fragments mapped (FRKM) was used as the indicator of the gene expression level. DESeq2 was used for identifying differentially expressed genes (DEGs) (Love et al., 2014). Fold change (FC) ≥ 2.0 and false discovery rate (FDR) < 0.01 were used as the screening criteria of DEGs.

### Quantitative RT-PCR

Reverse transcription was conducted using the FastQuant RT Super Mix (TIAN-GEN, China). The qPCR amplification was performed by using the 2 × SYBR Green qPCR Master Mix (US Everbright^®^ Inc., Suzhou, China) on a CFX96 Touch Deep Well^TM^ Real-Time PCR Detection System (Bio-Rad, United States) with three biological replications. Differential gene expression was calculated using the 2^–ΔΔCt^ method. The *BoActin1* was used as an internal reference control. Primer sequences for all genes are available in Supplementary Table 1.

### Functional Marker Development

According to the genome sequence of the interested region, a common polymorphic site between two parents and between extreme pools was developed for a molecular marker, of which the primers were 5′-TACACACGCTGATATACCAACA-3′ (forward) and 5′-TACACACGCTGCCCTGGAAA-3′ (reverse). The PCR amplification was conducted among *B. oleracea* lines in group II. The PCR products were separated into 1.5% agarose gels and visualized under UV light.

## Results

### Clubroot Resistance of Parents and Extreme Pools

Clubroot resistance was investigated in two parental lines and the two F2 groups across 2 years (Supplementary Table 2 and Figure 1B). When tested together with group I (the 1st year), “GZ87” exhibited a high resistance level to *P. brassicae* (mean DI of 6.3), while “263” showed an average DI value of 40 (Figure 1A). The two parental lines exhibited obvious differences in DI when tested together with group II (DI_GZ87_ = 0 and DI_263_ = 56.3). Each of 17 and 20 lines was selected from the 110 and 74 F2 vegetative lines in group I and group II, respectively, presenting an average DI of 10.6 and 57.4 in the R and the S pools, respectively, in group I and 1.3 and 49.0 in the two pools in group II (Table 1).

**TABLE 1:** Clubroot resistance of parental lines and extreme pools in groups I and II.

**ID**	**Group I**		**Group II**	
	**DI**	**DIC (%)**	**DI**	**DIC (%)**
GZ87	6.25 ± 0.82	25.00 ± 3.42	0.00 ± 0.00	0.00 ± 0.00
263	39.97 ± 2.69	86.67 ± 3.65	56.25 ± 5.38	100.00 ± 0.00
R pool	7.19 ± 2.35	18.50 ± 6.07	1.58 ± 2.81	5.43 ± 9.51
S pool	57.41 ± 6.17	96.43 ± 4.00	50.69 ± 12.13	88.83 ± 15.80
				

### Identification of Quantitative Trait Loci

An average of 10.2 Gb clean data (over 20 × of the reference genome) was yielded for each parental line and extreme pool in group I, with a content: guanine-cytosine content (GC) content of 37.7% and Q30 > 92.2%. A range of 96.7–97.3% of the clean reads was aligned to the reference genome of *B. oleracea*. A total of 1,338,555 high-quality SNPs and 3,381,999 indels were detected between the parents and between two pools (Supplementary Table 3), while in group II, an average of 14.2 Gb clean data was yielded for each sample, with a GC content of 37.6% and Q30 > 91.26%. A range of 88.67–90.09% of the clean reads was aligned to the reference genome of *B. oleracea*, revealing 732,713 high-quality SNPs and 30,624,760 indels for the following QTL-Seq analysis (Supplementary Table 3).

**FIGURE 2:**
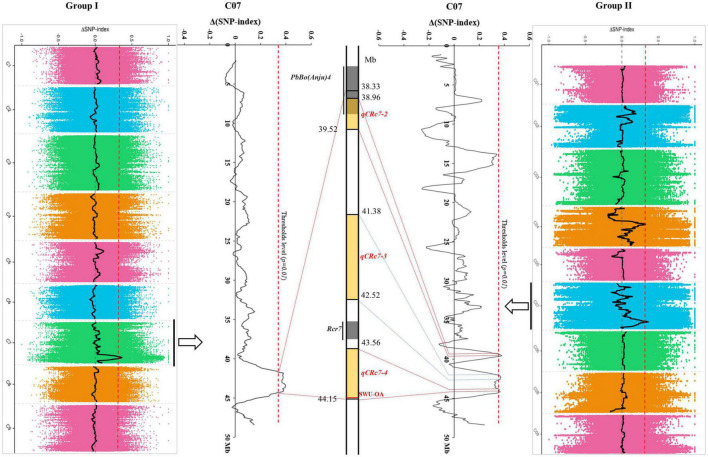
Comparison of clubroot resistance (CR)-quantitative trait loci (QTLs) identified by QTL-sequencing (QTL-seq) from group I **(left)** and group II **(right)**. The physical map of chromosome C07 (partial) was presented in the middle, with yellow backgrounds indicating QTLs identified in this study and gray backgrounds indicating loci identified in other studies.

After calculating Δ(SNP-index) of each locus and visualizing the Δ(SNP-index) trends by using the sliding window method within each group, 1/4 peaks were detected in group I/II with higher values than corresponding thresholds (Table 2). One QTL (*qCRc7-1*), in an interval from 38.33 to 44.14 Mb (5.81 Mb) on chromosome C07, was identified in group I. Four QTLs, one on chromosome C04 (*qCRc4-1*, 16.92–18.79 Mb) and three on chromosome C07 (*qCRc7-2*, 38.96–39.54 Mb; *qCRc7-3*, 41.38–42.52 Mb; *qCRc7-4*, 43.56–44.15 Mb) (Table 2), were detected in group II. Comparing QTLs between two groups, the three QTLs detected in group II were all located within the locus *qCRc7-1* identified in group I (Figure 2). Therefore, the three QTLs (i.e., *qCRc7-2*, *qCRc7-3*, and *qCRc7-4*) were focused on the following analysis.

**TABLE 2:** The CR-quantitative trait loci (QTLs) identified in groups I and II.

**QTL**	**Group**	**Chr**	**Confidence interval (Mb)**		**Interval (Mb) (Mb)**	**No. of SNP**	**No. of Indel**	**No. of genes**	**No. of DEGs**
			**Start**	**End**					
*qCRc7-1*	Group I	C07	38.33	44.14	5.81	28,284	19,830	698	123
*qCRc4-1*	Group II	C04	16.92	18.79	1.87	4,384	1,774	123	11
*qCRc7-2*	Group II	C07	38.96	39.52	0.56	4,300	2,117	79	16
*qCRc7-3*	Group II	C07	41.38	42.52	1.14	6,210	3,462	134	29
*qCRc7-4*	Group II	C07	43.56	44.15	0.59	3,258	1,874	99	16
									

### Potential Candidate Genes for Clubroot Resistance

**FIGURE 3:**
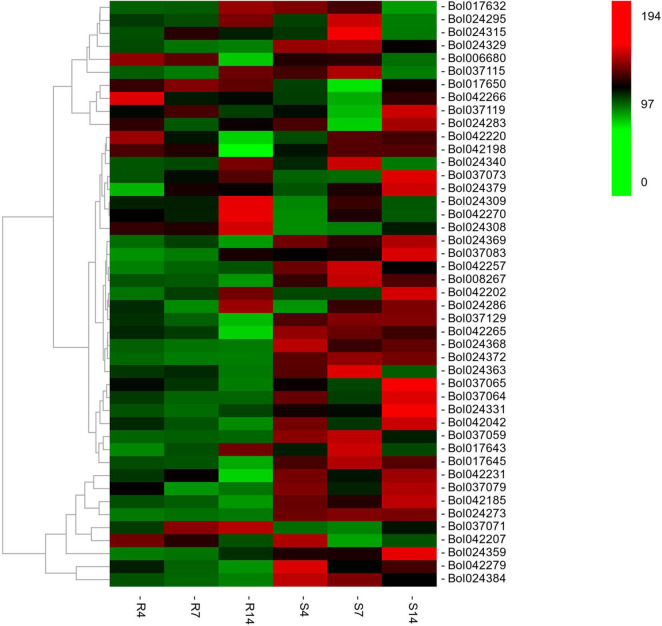
The heatmap analysis of the expression of 46 differentially expressed genes (DEGs) underlying the CIs of *qCRc4-1, qCRc7-2, qCRc7-3*, and *qCRc7-4*. The average expression values of genes in different inoculation times were denoted at the right with a color scale, in which green, black, and red color indicated the low, medium, and high level of expression, respectively.

**FIGURE 4:**
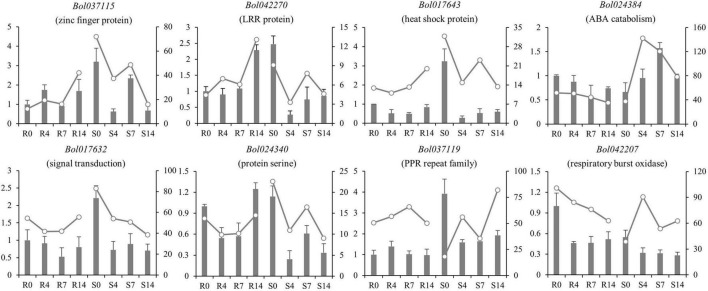
Comparison of expression patterns of nine DEGs located within C07 QTL regions in R and S pools before and after inoculation. The relative expression in qRT-PCR was indicated by bars with ordinate on the left; the FPKM in RNA-seq was presented by lines with ordinate on the right side.

The RNA-seq was conducted in R and S pools of group II to investigate the gene expression in three QTLs (i.e., *qCRc7-2*, *qCRc7-3*, and *qCRc7-4*). Over six Gb clean data were obtained from each sample (Q30 > 93%), of which 72.6–74.2% were aligned to the reference genome of *B. oleracea* (Supplementary Table 4). Compared to 0 DAI, 540/500/1837 and 2656/1518/4182 DEGs were detected from the R and the S pool at 4/7/14 DAI, respectively. In total, 11/16/28/16 DEGs were detected from 123/79/134/99 genes with large amounts of SNPs and indels located within the CIs of *qCRc4-1/qCRc7-2/qCRc7-3*/*qCRc7-4* (Table 2 and Supplementary Table 5). Then, 45 DEGs with high expression levels (the selection criterion is the average expression of samples with three replicates > 5) among the 71 DEGs in the CIs were selected for further screening of candidate genes. According to the heatmap analysis of the 45 DEGs, the expression levels of most genes in the S pool were higher than those in the R pool at the 4, 7, and 14 DAI, indicating that the response to *P. brassicae* may be more dramatic in S pool (Figure 3). Within these 45 DEGs, eight DEGs exhibiting obvious differential expression patterns between the R and the S pool were found to be located in the overlap regions on chromosome C07. The qRT-PCR revealed consistent expression patterns with RNA-seq for six genes among these eight genes (Figure 4). Among the six genes, four genes (i.e., *Bol017643*, *Bol017632, Bol024384*, and *Bol024340*) were generally not induced by the pathogen in the R pool, and only two genes (i.e., *Bol037115* and *Bol042270*) were upregulated after inoculation in the R pool but downregulated in the S pool. Of these, *Bol037115* [annotated as an FCS-like zinc finger (FLZ) protein] was located in *qCRc7-2*, with seven SNPs and one indel between two pools, and *Bol042270* [plant intracellular Ras-group-related leucine-rich repeat sequences (LRR) protein 8] was located in *qCRc7-4*, with nine SNPs and six indels between the two pools. The two genes were considered as potential candidates for future certification.

### Functional Marker for Clubroot Resistance

**FIGURE 5:**
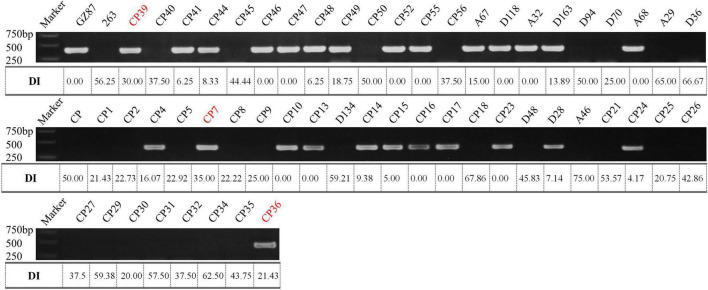
The PCR amplification of SWU-OA in two parents and F2 lines. Mismatches between the PCR products and the DI values were indicated in red.

According to the deep comparison between two parents and between extreme pools, a PCR primer pair named “SWU-OA” was designed within the interval of *qCRc7-4* (Figure 2). After the amplification by SWU-OA in 54 F2 lines in group II, 28 lines that exhibited the absence of the same bands (400 bp) as the R parent GZ87 were all S to the pathogen, with DI values ranging from 20.0 to 75.0 (averaged in 43.2), while 26 lines that presented the 400-bp bands were found to be with DI values of 0–18.6 (averaged in 4.2), with three exceptions that exhibited intermediate DI values (21.4–35.0) (Figure 5). It is indicated that the SWU-OA exhibited ∼95% accuracy in identifying CR in 56 F2 lines and possibly provided a way to accelerate the breeding process of *B. oleracea* with CR.

## Discussion

In the past, QTLs were mainly identified based on the genetic linkage map constructed by molecular markers, such as random amplified polymorphic DNA (RAPD), restriction fragment length polymorphism (RFLP), amplified fragment length polymorphism (AFLP), and simple-sequence repeats (SSR) markers (Landry et al., 1992; Suwabe et al., 2006; Nagaoka et al., 2010), which is usually time-consuming (involves labor-intensive genotyping work). With the rapid development of sequencing technology in recent years, many researchers have combined bulked segregation analysis (BSA) with the whole genome and transcriptome to search for candidate genes (Dakouri et al., 2018; Zhou et al., 2020). With the complement of *B. oleracea* genome information, the QTL-Seq approach (Takagi et al., 2013) combined with transcriptome sequencing made it easier to identify potential candidate genes for traits of interest.

As compared with that of *B. rapa* and *B. napus*, fewer CR loci were identified from *B. oleracea* possibly due to the lack of R resources. The reported CR loci of *B. oleracea* were detected from chromosomes C01 (*Pb3, PbBo1*), C02 [*Pb-Bo(Anju)1*, *Pb-Bo(Anju)2, CRQTL-YC*], C03 [*Pb-Bo(Anju)3, Pb4*], C07 [*Rcr7, Pb-Bo(Anju)4*], and C09 (*CRQTL-GN_1*, *CRQTL-GN_2*) (Voorrips et al., 1997; Moriguchi et al., 1999; Rocherieux et al., 2004; Nagaoka et al., 2010; Lee et al., 2015; Dakouri et al., 2018). In our study, we identified four QTLs for CR, of which one located on chromosome C04 and the other three located adjacently on C07. No CR-QTL has been detected before on C04 in *B. oleracea*, but two CR-QTLs (*SCR-C4a* and *SCR-C4b*) were found from chromosome C04 in *B. napus* (Li et al., 2016). By aligning sequences of the markers in *SCR-C4a* and *SCR-C4b* to the reference genome of *B. oleracea*, the two QTLs were aligned to 2.49–2.51 Mb and 8.06–8.10 Mb on C04 of *B. oleracea*, which were obviously distant from the interval of our *qCRc4-1* (16.92–18.79 Mb). By using the same approach, CR-QTLs on chromosome C07 were compared among studies in both *B. oleracea* and *B. napus*. In *B. oleracea*, the CR loci *PbBo(Anju)4* (Nagaoka et al., 2010) was found to be located in 37.93–39.25 Mb, which was partially overlapped with our *qCRc7-2* (38.96–39.52 Mb). However, *qCRc7-2* may have a limited effect on CR since *PbBo(Anju)4* was reported to be with a very small effect (*R*^2^ = 0.03), and we failed in finding the candidate resistance gene from this region. The other two QTLs for CR on C07, i.e., *qCRc7-3* (41.38–42.52 Mb) and *qCRc7-4* (43.56–44.15 Mb), were found to be located nearby but not overlapped with *Rcr7* (42.94–43.20 Mb), which is a major QTL in *B. oleracea* for resistance against *P. brassicae* pathotypes 3 and 5X (Dakouri et al., 2018). In addition, the most possible candidate gene for *Rcr7* (*Bo7g108760*, a TIR-NBS-LRR disease resistance gene) (Dakouri et al., 2018) was not induced by *P. brassicae* race 4 in this study (FPKM of 0.5, 0.6, 0.4, and 0.4 in R0, R4, R7, and R14; 1.6, 0.5, 0.8, and 0.6 in S0, S4, S7, and S14, respectively), whereas, an SNP association analysis in *B. napus* detected a significant locus from chromosome C07 at 42.02–42.22 Mb, which is located within the homologous region of our *qCRc7-3*. These suggest that chromosome C07 of *B. oleracea* possibly carries multiple QTLs for CR, and *qCRc7-4* is probably a novel CR locus.

In a previous study, 23 QTLs for three CR-associated traits were identified from the same segregating population between “GZ87” and “263,” including DIC, numbers of fibrous roots (NFR), and *P. brassicae* content in roots (PCR) (Peng et al., 2018). Of these, four QTLs (i.e., *NFR.I-3*, *NFR.I-4*, *NFR.II-4*, and *PCR.II-3*) were located on chromosome C04 and two QTLs (i.e., *NFR.II-6* and *NFR.II-7*) were located on chromosome C07. However, after a comparison of the physical positions of these loci, none of them was overlapped with the QTLs for CR found in this study. The closest loci between the two studies were *NFR.II-6* (47.77–48.30 Mb) and *qCRc7-4*, which showed a distance of 3.62 Mb on chromosome C07. This suggests that DIC, NFR, and *P. brassicae* content in roots may not be representative indicators for CR, which is usually determined by DI.

A total of 312 genes were found to be located in the three QTL regions on C07, including six R genes encoding TIR-NBS-LRR disease resistance proteins. However, none of these R genes presented an expression difference between R and S pools in RNA-seq (Supplementary Table 6). Although 61 DEGs were identified from the three regions, most of them presented similar expression patterns between the two pools excepting eight genes. Among these, an FLZ domain protein (*Bol037115*) and a plant intracellular Ras-group-related LRR (PIRL) protein (*Bol042270*) exhibited over threefold upregulation after inoculation in the R pool but with downregulation in the S pool. The FLZ domain proteins are implicated in the regulation of various biotic and abiotic stresses (Chen X. et al., 2013; Jamsheer and Laxmi, 2015). PIRLs encode a plant-specific class of leucine-rich repeat proteins related to Ras-interacting LRRs that take part in developmental signaling in animals and fungi (Forsthoefel et al., 2005). It seems that both the two candidate genes are potentially involved in response to hormones, which are tightly associated with host response to pathogens. Nevertheless, further studies are needed to validate the roles of these two genes in CR, as well as unravel their resistance mechanisms.

Resistant varieties are of great importance in the control of clubroot of *Brassica* crops, but breeding in *B. oleracea* has been largely unsuccessful due to the lack of highly R sources and the complexity of this quantitative trait (Laurens and Thomas, 1993; Nagaoka et al., 2010; Tomita et al., 2013). Although a few QTLs have been identified in *B. oleracea*, the effects of these QTLs were usually not as high as expected, and usable markers for marker-assisted selection (MAS) were quite limited recently. For example, Tomita et al. (2013) identified several QTLs for CR in *B. oleracea*, but the major QTL *PbBo(Anju)1* was not enough to produce sufficient resistance against *P. brassicae*, while the genotype contained five CR-QTLs produced high resistance; Nomura et al. (2005) identified three CR-QTLs from the cross between a cabbage and a kale line and found that the cumulation of those three QTLs showed high resistance to three isolates of *P. brassicae*, whereas the mean DI in the plants carrying only single QTL was intermediate. Therefore, multigene pyramiding breeding centering on MAS is necessary for the breeding of R varieties in *B. oleracea*. In our study, several QTLs were identified, and a molecular marker (SWU-OA) that developed from the polymorphic region within *qCRc7-4* was effective in distinguishing the R or S F2 lines (with an accuracy of 95%). This suggested a great potential of this marker to be applied in MAS of offspring with CR, as well as in the pyramiding of our QTL with other CR-QTLs to create new *B. oleracea* resources. This is of practical significance in breeding of high R *B. oleracea* varieties with multiple CR-genes (QTLs).

## Data Availability Statement

The datasets presented in this study can be found in online repositories. The names of the repository/repositories and accession number(s) can be found below: NCBI, BioProject ID: PRJNA735118.

## Author Contributions

JS, HS, and JM designed and directed the experiments. FC, HH, YZ, WH, QL, and XR performed most of the experiments and analyzed the data. JM and FC wrote the manuscript. JS, HS, FY, and WQ discussed the results and improved the manuscript. All authors contributed to the article and approved the submitted version.

## Conflict of Interest

The authors declare that the research was conducted in the absence of any commercial or financial relationships that could be construed as a potential conflict of interest.

## Publisher’s Note

All claims expressed in this article are solely those of the authors and do not necessarily represent those of their affiliated organizations, or those of the publisher, the editors and the reviewers. Any product that may be evaluated in this article, or claim that may be made by its manufacturer, is not guaranteed or endorsed by the publisher.

## Footnotes

1
http://39.100.233.196:82/download_genome/Brassica_Genome_data/Braol_JZS_V1.1/BOL.seq.lst.new.chr20110802_check.fa.gz

## References

[B1] Chang A. Lamara M. Wei Y. Hu H. Parkin I. Gossen B. (2019). Clubroot resistance gene *Rcr6* in *Brassica nigra* resides in a genomic region homologous to chromosome A08 in *B. rapa*. *BMC Plant Biol.* 19:224. 10.1186/s12870-019-1844-5 31142280 PMC6542104

[B2] Chen J. Jing J. Zhan Z. Zhang T. Zhang C. Piao Z. (2013). Identification of novel QTLs for isolate-specific partial resistance to *Plasmodiophora brassicae* in *Brassica rapa*. *PLoS One* 8:e85307. 10.1371/journal.pone.0085307 24376876 PMC3869933

[B3] Chen X. Zhang Z. Visser R. G. F. Broekgaarden C. Vosman B. (2013). Overexpression of *IRM1* enhances resistance to aphids in *Arabidopsis thaliana*. *PLoS One* 8:e70914. 10.1371/journal.pone.0070914 23951039 PMC3741364

[B4] Chu M. Song T. Falk K. C. Zhang X. Liu X. Chang A. (2014). Fine mapping of *Rcr1* and analyses of its effect on transcriptome patterns during infection by *Plasmodiophora brassicae*. *BMC Genomics* 15:1166. 10.1186/1471-2164-15-1166 25532522 PMC4326500

[B5] Dakouri A. Zhang X. Peng G. Falk K. C. Gossen B. D. Strelkov S. E. (2018). Analysis of genome-wide variants through bulked segregant RNA sequencing reveals a major gene for resistance to *Plasmodiophora brassicae* in *Brassica oleracea*. *Sci. Rep.* 8:17657. 10.1038/s41598-018-36187-5 30518770 PMC6281628

[B6] Devos S. Vissenberg K. Verbelen J.-P. Prinsen E. (2005). Infection of Chinese cabbage by *Plasmodiophora brassicae* leads to a stimulation of plant growth: impacts on cell wall metabolism and hormone balance. *New Phytol.* 166 241–250. 10.1111/j.1469-8137.2004.01304.x 15760367

[B7] Dixon G. R. (2009). The occurrence and economic impact of *Plasmodiophora brassicae* and clubroot disease. *J. Plant Growth Regul.* 28 194–202.

[B8] Doyle J. (1991). “DNA protocols for plants,” in *Molecular Techniques in Taxonomy*, eds Hewitt G. M. Johnston A. W. B. Young J. P. W. (Berlin: Springer), 283–293.

[B9] Fekih R. Takagi H. Tamiru M. Abe A. Natsume S. Yaegashi H. (2013). MutMap+: genetic mapping and mutant identification without crossing in rice. *PLoS One* 8:e68529. 10.1371/journal.pone.0068529 23874658 PMC3707850

[B10] Forsthoefel N. R. Cutler K. Port M. D. Yamamoto T. Vernon D. M. (2005). PIRLs: a novel class of plant intracellular leucine-rich repeat proteins. *Plant Cell Physiol.* 46 913–922. 10.1093/pcp/pci097 15809230

[B11] Friberg H. Lagerlöf J. Rämert B. (2005). Germination of *Plasmodiophora brassicae* resting spores stimulated by a non-host plant. *Eur. J. Plant Pathol.* 113:275. 10.1007/s10658-005-2797-0

[B12] Hejna O. Havlickova L. He Z. Bancroft I. Curn V. (2019). Analysing the genetic architecture of clubroot resistance variation in *Brassica napus* by associative transcriptomics. *Mol. Breed.* 39:112. 31396013 10.1007/s11032-019-1021-4 PMC6647481

[B13] Huang Z. Peng G. Gossen B. Yu F. (2019). Fine mapping of a clubroot resistance gene from turnip using SNP markers identified from bulked segregant RNA-Seq. *Mol. Breed.* 39:131. 10.1007/s11032-019-1038-8 PMC5581393 28894454

[B14] Huang Z. Peng G. Liu X. Deora A. Falk K. Gossen B. (2017). Fine mapping of a clubroot resistance gene in chinese cabbage using SNP markers identified from bulked segregant RNA sequencing. *Front. Plant Sci.* 8:1448. 10.3389/fpls.2017.01448 28894454 PMC5581393

[B15] Jamsheer K. M. Laxmi A. (2015). Expression of *Arabidopsis FCS-Like Zinc finger* genes is differentially regulated by sugars, cellular energy level, and abiotic stress. *Front. Plant Sci.* 6:746. 10.3389/fpls.2015.00746 26442059 PMC4585328

[B16] Karim M. M. Dakouri A. Zhang Y. Chen Q. Peng G. Strelkov S. (2020). Two clubroot-resistance genes, *Rcr3* and *Rcr9wa*, mapped in *Brassica rapa* using bulk segregant RNA sequencing. *Int. J. Mol. Sci.* 21:5033. 10.3390/ijms21145033 32708772 PMC7404267

[B17] Kuginuki Y. Ajisaka H. Yui M. Yoshikawa H. Hida K.-I. Hirai M. (1997). RAPD markers linked to a clubroot-resistance locus in *Brassica rapa* L. *Euphytica* 98 149–154. 10.1023/A:1003147815692

[B18] Landry B. Hubert N. Crete R. Chang M. Lincoln S. Etoh T. (1992). A genetic map for *Brassica oleracea* based on RFLP markers detected with expressed DNA sequences and mapping of resistance genes to race 2 of *Plasmodiophora brassicae* (Woronin). *Genome* 35 409–420. 10.1139/g92-061 33356898

[B19] Laurens F. Thomas G. (1993). Inheritance of resistance to clubroot (*Plasmodiophora brassicae* Wor.) in Kale (*Brassica oleracea* ssp. Acephala). *Hereditas* 119 253–262. 10.1111/j.1601-5223.1993.00253.x

[B20] Lee J. Izzah N. Choi B.-S. Joh H. Lee S.-C. Perumal S. (2015). Genotyping-by-sequencing map permits identification of clubroot resistance QTLs and revision of the reference genome assembly in cabbage (*Brassica oleracea* L.). *DNA Res.* 23 29–41. 10.1093/dnares/dsv034 26622061 PMC4755525

[B21] Li H. Durbin R. (2009). Fast and accurate short read alignment with Burrows–Wheeler transform. *Bioinformatics* 25 1754–1760. 10.1093/bioinformatics/btp324 19451168 PMC2705234

[B22] Li L. Luo Y. Chen B. Xu K. Zhang F. Li H. (2016). A genome-wide association study reveals new loci for resistance to clubroot disease in *Brassica napus*. *Front. Plant Sci.* 7:1483. 10.3389/fpls.2016.01483 27746804 PMC5044777

[B23] Liu Y. Xu A. Liang F. Yao X. Wang Y. Liu X. (2018). Screening of clubroot-resistant varieties and transfer of clubroot resistance genes to Brassica napus using distant hybridization. *Breed. Sci.* 68 258–267. 10.1270/jsbbs.17125 29875610 PMC5982190

[B24] Love M. I. Huber W. Anders S. (2014). Moderated estimation of fold change and dispersion for RNA-seq data with DESeq2. *Genome Biol.* 15:550. 10.1186/s13059-014-0550-8 25516281 PMC4302049

[B25] Ludwig-Müller J. Prinsen E. Rolfe S. A. Scholes J. D. (2009). Metabolism and plant hormone action during clubroot disease. *J. Plant Growth Regul.* 28 229–244. 10.1007/s00344-009-9089-4

[B26] Luo P. Lan Z. Deng J. Wang Z. (2000). Application of in vitro organ culture in wide-cross breeding of rapeseed. *Euphytica* 114 217–221. 10.1023/A:1003911507540

[B27] Manzanares-Dauleux M. J. Delourme R. Baron F. Thomas G. (2000). Mapping of one major gene and of QTLs involved in resistance to clubroot in Brassica napus. *Theor. Appl. Genet.* 101 885–891. 10.1007/s001220051557

[B28] Mehraj H. Akter A. Miyaji N. Miyazaki J. Shea D. Fujimoto R. (2020). Genetics of clubroot and fusarium wilt disease resistance in brassica vegetables: the application of marker assisted breeding for disease resistance. *Plants* 9:726. 10.3390/plants9060726 32526827 PMC7355935

[B29] Moriguchi K. Kimizuka-Takagi C. Ishii K. Nomura K. (1999). A genetic map based on RAPD, RFLP, isozyme, morphological markers and QTL analysis for clubroot resistance in *Brassica oleracea*. *Breed. Sci.* 49 257–265. 10.1270/jsbbs.49.257 26081539

[B30] Nagaoka T. Doullah M. A. U. Matsumoto S. Kawasaki S. (2010). Identification of QTLs that control clubroot resistance in *Brassica oleracea* and comparative analysis of clubroot resistance genes between B. rapa and B. oleracea. *Theor. Appl. Genet.* 120 1335–1346. 10.1007/s00122-010-1259-z 20069415

[B31] Navabi Z.-K. Huebert T. Sharpe A. G. O’Neill C. M. Bancroft I. Parkin I. A. P. (2013). Conserved microstructure of the *Brassica* B Genome of *Brassica nigra* in relation to homologous regions of *Arabidopsis thaliana*, *B. rapa* and *B. oleracea*. *BMC Genomics* 14:250. 10.1186/1471-2164-14-250 23586706 PMC3765694

[B32] Nomura K. Minegishi Y. Kimizuka-Takagi C. Fujioka T. Moriguchi K. Shishido R. (2005). Evaluation of F2 and F3 plants introgressed with QTLs for clubroot resistance in cabbage developed by using SCAR markers. *Plant Breed.* 124 371–375. 10.1111/j.1439-0523.2005.01105.x

[B33] Peng G. Pageau D. Strelkov S. E. Gossen B. D. Hwang S.-F. Lahlali R. (2015). A >2-year crop rotation reduces resting spores of *Plasmodiophora brassicae* in soil and the impact of clubroot on canola. *Eur. J. Agron.* 70 78–84. 10.1016/j.eja.2015.07.007

[B34] Peng L. Zhou L. Li Q. Wei D. Ren X. Song H. (2018). Identification of Quantitative trait loci for clubroot resistance in *Brassica oleracea* with the use of brassica SNP microarray. *Front. Plant Sci.* 9:822. 10.3389/fpls.2018.00822 29967632 PMC6015909

[B35] Rocherieux J. Glory P. Giboulot A. Boury S. Barbeyron G. Thomas G. (2004). Isolate-specific and broad-spectrum QTLs are involved in the control of clubroot in *Brassica oleracea*. *Theor. Appl. Genet.* 108 1555–1563. 10.1007/s00122-003-1580-x 15007504

[B36] Strelkov S. E. Tewari J. P. Smith-Degenhardt E. (2006). Characterization of *Plasmodiophora brassicae* populations from Alberta, Canada. *Can. J. Plant Pathol.* 28 467–474. 10.1080/07060660609507321

[B37] Suwabe K. Tsukazaki H. Iketani H. Hatakeyama K. Kondo M. Fujimura M. (2006). Simple sequence repeat-based comparative genomics between *Brassica rapa* and *Arabidopsis thaliana*: the genetic origin of clubroot resistance. *Genetics* 173 309–319. 10.1534/genetics.104.038968 16723420 PMC1461432

[B38] Takagi H. Abe A. Yoshida K. Kosugi S. Natsume S. Mitsuoka C. (2013). QTL-seq: rapid mapping of quantitative trait loci in rice by whole genome resequencing of DNA from two bulked populations. *Plant J.* 74 174–183. 10.1111/tpj.12105 23289725

[B39] Tomita H. Shimizu M. Asad-ud Doullah M. Fujimoto R. Okazaki K. (2013). Accumulation of quantitative trait loci conferring broad-spectrum clubroot resistance in *Brassica oleracea*. *Mol. Breed.* 32 889–900. 10.1007/s11032-013-9918-9

[B40] Voorrips R. Jongerius M. Kanne H. (1997). Mapping of two genes for resistance to clubroot (*Plasmodiophora brassicae*) in a population of doubled haploid lines of *Brassica oleracea* by means of RFLP and AFLP markers. *Theor. Appl. Genet.* 94 75–82. 10.1007/s001220050384 19352748

[B41] Werner S. Diederichsen E. Frauen M. Schondelmaier J. Jung C. (2007). Genetic mapping of clubroot resistance genes in oilseed rape. *Theor. Appl. Genet.* 116:363. 10.1007/s00122-007-0674-2 18040658

[B42] Yu F. Zhang X. Huang Z. Chu M. Song T. Falk K. (2016). Identification of genome-wide variants and discovery of variants associated with *Brassica rapa* clubroot resistance gene *Rcr1* through bulked segregant RNA sequencing. *PLoS One* 11:e0153218. 10.1371/journal.pone.0153218 27078023 PMC4831815

[B43] Yu F. Zhang X. Peng G. Falk K. C. Strelkov S. E. Gossen B. D. (2017). Genotyping-by-sequencing reveals three QTL for clubroot resistance to six pathotypes of *Plasmodiophora brassicae* in *Brassica rapa*. *Sci. Rep.* 7:4516. 10.1038/s41598-017-04903-2 28674416 PMC5495781

[B44] Zhou Q. Galindo Gonzalez L. Hwang S. Strelkov S. (2020). Application of genomics and transcriptomics to accelerate development of clubroot resistant canola. *Can. J. Plant Pathol.* 43 189–208. 10.1080/07060661.2020.1794541

[B45] Zhou Z. Jiang Y. Wang Z. Gou Z. Lyu J. Li W. (2015). Resequencing 302 wild and cultivated accessions identifies genes related to domestication and improvement in soybean. *Nat. Biotechnol.* 33 408–414. 10.1038/nbt.3096 25643055

[B46] Zhu H. Zhai W. Li X. Zhu Y. (2019). Two QTLs controlling clubroot resistance identified from bulked segregant sequencing in pakchoi (*Brassica campestris* ssp. *chinensis* Makino). *Sci. Rep.* 9:9228. 10.1038/s41598-019-44724-z 31239512 PMC6592919

